# Chromatin Function Modifying Elements in an Industrial Antibody Production Platform - Comparison of UCOE, MAR, STAR and cHS4 Elements

**DOI:** 10.1371/journal.pone.0120096

**Published:** 2015-04-07

**Authors:** Fay Saunders, Berni Sweeney, Michael N. Antoniou, Paul Stephens, Katharine Cain

**Affiliations:** 1 Protein Expression and Purification Group, UCB, Slough, Berkshire, United Kingdom; 2 Gene Expression and Therapy Group, King’s College School of Medicine, Department of Molecular Genetics, Guy’s Hospital, London Bridge, London, United Kingdom; National Cancer Institute, NIH, UNITED STATES

## Abstract

The isolation of stably transfected cell lines suitable for the manufacture of biotherapeutic protein products can be an arduous process relying on the identification of a high expressing clone; this frequently involves transgene amplification and maintenance of the clones’ expression over at least 60 generations. Maintenance of expression, or cell line stability, is highly dependent upon the nature of the genomic environment at the site of transgene integration, where epigenetic mechanisms lead to variable expression and silencing in the vast majority of cases. We have assessed four chromatin function modifying elements (A2UCOE, MAR X_S29, STAR40 and cHS4) for their ability to negate chromatin insertion site position effects and their ability to express and maintain monoclonal antibody expression. Each element was analysed by insertion into different positions within a vector, either flanking or between heavy chain (HC) and light chain (LC) antibody expression cassettes. Our results clearly show that the A2UCOE is the most beneficial element in this system, with stable cell pools and clones increasing antibody yields 6.5-fold and 6.75-fold respectively. Stability analysis demonstrated that the reduction in antibody expression, seen with cells transfected with the control vector over 120 generations, was mitigated in the clones containing A2UCOE-augmented transgenes. Analysis also showed that the A2UCOE reduced the amount of transgene promoter DNA methylation, which contributed to the maintenance of starting levels of expression.

## Introduction

Monoclonal antibodies (mAb) are a significant component of the therapeutic biopharmaceutical industry due to their high specificity and ability to elicit an immune response against virtually any target. Currently 25 therapeutic monoclonal antibodies are being produced in mammalian cells with the Chinese hamster ovary (CHO) cell line being the preferred choice as their products are both bioactive and safe in humans [1]. Generation of stably transfected cell lines expressing therapeutic protein products follows a defined multistep process that begins with vector design and ends with a clonal cell line with optimal characteristics for manufacturing. Characteristics must include high levels of productivity and long-term stability (at least 60 generations) of transgene expression, in the absence of antibiotic drug selective pressure beyond the production of a manufacturing working cell bank [2]. A number of studies have shown that the site of transgene integration greatly influences its expression and is a factor that determines how the cell line behaves with regard to prolonged stability [3–5]. The generation of stably transfected cells usually begins with random integration of a gene of interest (GOI) into the target cell genome. However, since in any given cell type the majority of the genome consists of transcriptionally non-permissive heterochromatin, there is a high probability that the GOI will be integrated into an area which is unfavourable for high level and stable expression [6]. In addition, even if a GOI has integrated into a transcriptionally active region, expression may still be silenced due to DNA methylation within the integrated transgene or its promoter region [7–8]. These epigenetic processes can lead to either a variegated transgene expression pattern (known as position effect variegation, PEV) or complete silencing over time.

Linking certain types of genetic elements namely ubiquitous chromatin opening elements (UCOEs), Scaffold or Matrix Attachment Regions (S/MARs), Stabilising Anti Repressor (STAR) elements and insulators to the GOI can reduce or negate epigenetic processes from negatively affecting transgene expression even if the gene has been integrated into an area of closed heterochromatin [9–10].

UCOEs were initially identified in studies of the *TBP-PSMB1* and the *HNRPA2B1-CBX3* housekeeping gene loci [11]. It was discovered that transgenes containing the dual divergently transcribed promoter regions of these loci, which are encompassed within a methylation-free CpG island were able to confer stable expression even from within a centromeric heterochromatin environment demonstrating a dominant chromatin opening capability [11]. It was subsequently also shown that sub-fragments extending over the dual divergently transcribed promoter region of *HNRPA2B1-CBX3* were able to not only increase expression of a CMV-EGFP cassette by greater than 20-fold, but also negated silencing and maintained stability of expression over 100 generations in stably transfected pools and clones of CHO-K1 cells [12–13]. More recently it has been shown that the *HNRPA2B1* promoter of the *HNRPA2B1-CBX3* UCOE (designated as A2UCOE) can drive stable transgene expression both *in vitro* and in haematopoietic stem cells *in vivo* from within lentiviral gene therapy vectors [14]. Furthermore, the 1.5kb core A2UCOE [12] or a 1.2kb sub-fragment can confer stability of expression from linked ubiquitous [14–15] or tissue specific promoters [16–17].

Scaffold or Matrix Attachment Regions (S/MARs) were first identified in DNA fragments that were retained in nuclear scaffold/matrix preparations [18]. S/MARs have been shown to act as structural components, assist packaging of DNA in the nucleus, form genetic boundaries between chromosomal domains, provide insulation from chromatin effects, increase transcription initiation levels, and also have the potential to protect against position effects and enhance gene expression [9–10, 19–24]. The MAR used in our study, MAR X_S29, was identified in a bioinformatics search of the human genome and was shown to increase the level of CMV-GFP transgene expression when stably transfected into CHO cells [10]. MAR X_S29 is located on the chromosome X and is predicted to be part of an intron in the *leucine rich repeats and calponin homology domain containing 2* (*LRCH2*) gene.

Stabilising Anti Repressor (STAR) elements were identified during a screen of a library of human genomic DNA fragments for their ability to block chromatin associated repressors allowing transfected cells to survive in the presence of antibiotics due to negation of silencing of the linked resistance marker gene [25]. The prototypical STAR40 element, located on chromosome 22, at q11.1 upstream of the *IL17R* gene, consists of a unique non-coding DNA sequence lacking in CpG islands. Vectors containing GOIs driven by different promoters and flanked by the STAR40 element gave rise to greater numbers of stably transfected cell colonies and increased transgene expression levels proportional to transgene copy number [25].

DNaseI hypersensitive site 4 of the chicken beta-globin locus control region (cHS4) is an element showing classical insulator properties [26]. It has been demonstrated that cHS4 has both enhancer blocking [27] and barrier activity and contains a ‘core’ 250bp region made up of five protein factor binding sites, three of which are necessary for appropriate barrier activity [7, 27–29]. The functionality of the cHS4 insulator appears to be host cell dependent with effective insulator control observed in Drosophilia [27], human K562 erythroleukemia tissue culture cells [30–31], transgenic mice [32]) and oncoretroviral vectors [33]. However, it has been shown to be only partially effective at insulating the CMV promoter from position effects during transgene expression in CHO cells [34].

A recent study found that the A2UCOE, cHS4 and MAR elements could provide barrier function and reduce the spread of telemetric heterochromatin to a distally placed reporter gene [35]. In addition, the MAR and A2UCOE elements were found to be associated with distinct sets of histone modification marks representative of their respective chromatin remodelling and transcriptional activating capabilities.

We report here the first direct comparison of the UCOE, MAR, STAR and cHS4 chromatin modifying elements within the same vector context containing an industrially relevant heavy and light chain antibody expression system. The 1.5kb A2UCOE, MAR X_S29, STAR40 and cHS4 elements were incorporated into an antibody expression plasmid in different configurations. The optimal configuration for each element was determined by analysing their effects on antibody expression in both pools and clonal derivatives of stably transfected CHO-K1 cells. Our results show that of the four elements studies, the A2UCOE is the most effective at increasing yields in pools and clonal cell lines and also improved cell line expression stability compared to the control.

## Material and Methods

### Antibody expression vector construct generation

The 1.5 kb core A2UCOE [12], a 1kb STAR40 and a 0.5kb cHS4 elements were commercially synthesised (Entelechon, Regensburg, Germany) (S1 Fig). The MAR X_S29 was amplified as a 3.36kb fragment using a polymerase chain reaction procedure from the PAC human genomic clone RP4-736G20 (Welcome Trust Sanger Institute, Cambridge, UK). The oligonucleotides used to amplify the MAR X_S29 are shown in Table 1. All clones were generated incorporating *Mlu*I, *Not*I, and *Xho*I restriction enzyme sites at their 5’ end and *Asc*I, *Eag*I and *Sal*I sites at their 3’ end. The control plasmid was generated by cloning the immunoglobulin heavy chain (HC) variable region into a mammalian expression vector pMmouseIgG1 (UCB) as a *HindIII*-*Xho*I fragment and the light chain (LC) variable region into a mammalian expression vector pMmousecK (UCB) as a *Hind*III-*BsiW*I fragment. These two plasmids were double digested with *Sal*I and *Not*I and the relevant fragments excised and ligated to generate a vector containing both the HC and LC antibody genes. The A2UCOE, STAR40, cHS4 and MAR X_S29 elements were then incorporated into the control vector using restriction enzyme digestion and ligation. Briefly, a *Sal*I digest allowed for the introduction of the various elements 3’ of the HC cassette, and a *Mlu*I digest for insertion at the 5’ end of the LC gene, or a *NotI* digest for a 5’ insertion to HC. The A2UCOE, STAR40, cHS4 and MAR X_S29 elements were digested with *Sal*I + *Xho*I, or *Mlu*I + *Asc*I or *Not*I + *Eag*I, which generated fragments with compatible ends to those of the antibody vector digests and ligated accordingly. The second copy of the A2UCOE, STAR40, cHS4 or MAR X_S29 element was subsequently cloned into either the 3’HC or the 5’LC vectors depending on selected configuration. Restricting the 3’HC vector with *Not*I and ligating in a fragment of DNA with compatible ends generated the vector 3’HC 5’HC. Restricting the 3’HC and the 5’LC vectors with *Mlu*I and ligating in a fragment with compatible ends generated the vectors 3’HC 5’LC and 5’LC 3’HC respectively. To generate a triple element vector (3’HC 5’LC 5’HC) the DNA fragments were cloned into the *Not*I site of the 3’HC 5’LC vector. The *neomycin phosphotransferase* (*neo*) gene was cloned into all the vectors as a *Sal*I/*Xho*I fragment. To introduce the *glutamine synthetase* (*GS*) gene the *neo* cassette was exchanged with a *Sal*I-*Mlu*I fragment containing the GS minigene cassette.

**Table 1:** Oligonucleotides and probes.

**MARX_S29 PCR**	
MAR X_S29 Forward (MAR F)	CGGCCGCACGCGTCTCGAGGATCCCTTTATAAAACCAC
MAR X_S29 Reverse (MAR R)	GTCGACGGCGCGCCCGGCCGATGGATCCCTATGCTGCTGGTTTACAG
**TaqMan Probes**	
Hamster GAPDH Forward	CTGCCACCCAGAAGACTGT
Hamster GAPDH Reverse	GTGGATGCAGGGATGATGTTCT
Hamster GAPDH Reporter	ATCACGCCACAGCTTT
Murine Kappa Forward	GGAAGATTGATGGCAGTGAACGA
Murine Kappa Reverse	GCTGTCCTGATCAGTCCAACT
Murine Kappa Reporter	TCAGGACGCCATTTTG
Murine Heavy Forward	GAGCAGTTCAACAGCACTTTCC
Murine Heavy Reverse	GCCAGTCCTGGTGCATGAT
Murine Heavy Reporter	CAGTCAGTGAACTTCC
**Southern blot hybridization probe generation**	
Forward	GTCATGAGATTATCAAAAAGGATC
Reverse	CGCCTTGATCGTTGGGAACC
**Bisulphite sequencing**	
BSPLF1	TTATAGGTGTGGGTTATTGATT
BSPLR1	AAAAAATAACCACCCTAACAAT
BSPLF2	TTTTTATAGGATGGGGTTTTATT
BSPLR2	CACATCTAACACCTAAAAACCA
BSPHF1	GTTATATTGTTTTTGGTTTGGG
BSPHR1	TACTCCAAACCCTTCTCAAATAC
BSPHF2	TTTTGTATTTTTATAGGATGGGGT
BSPHR2	ACTCCCTCCAAATTTAACAAAC

Sequence of primers used for generation of the MARX_S29 fragment by PCR, quantitative PCR for determining GAPDH, HC and LC gene expression employing the TaqMan system, generation of transgene Southern blot hybridisation probes, and PCR amplification of CMV genomic products following sodium bisulphite convertion.

### Generation of pool and stable cell lines

Pools of stably transfected CHO-K1 (UCB Pharma) cells were generated using Lipofectamine 2000 (Life Technologies Ltd, Paisley, UK) and selected using geneticin sulphate (G418). A total of 1x10^7^ cells were transfected according to the manufacturer’s protocol. Stably transfected cells were then selected for by addition of 1.5mg/ml G418. Fetal calf serum (FCS; PAA Laboratories Ltd, Yeovil, UK) was added to 1% (v/v) to allow surviving cells to adhere and medium changed after one week. At two weeks post transfection cells were transferred to shaking flasks and cultures set up to assess antibody expression levels. The generation of stably transfected pools of CHO-K1 cells under GS selection was by electroporation. Cells (1x10^7^) in 700μl CD-CHO medium (700μl) were mixed with 100μl of linearised antibody expressing plasmid DNA with or without a chromatin modifying element (at a concentration of 400μg/ml) in a Bio-Rad 0.4cm electroporation cuvette (Bio-Rad Laboratories Ltd, Hemel Hempstead, UK). The cell/DNA mixture was electroporated with a single pulse of 300 volts and 960μF using a Bio-Rad Gene Pulsar instrument (Bio-Rad Laboratories Ltd). The cell suspension was carefully removed from the cuvette and placed into 50ml of warm CD-CHO medium. The cells were incubated overnight at 37°C in a static T175 flask. Stable transfectants were then selected for by addition of 50μM methionine sulphoximine (MSX) to the media 24 hours post transfection. After 10 days the cell suspension was centrifuged at 1,000rpm and the cells resuspended in fresh CD-CHO medium supplemented with 50μM MSX or 1mg/ml G418. Once cell viability and number had recovered, cultures were set up to assess antibody expression levels.

Clonal cell lines were isolated by plating the pooled stable cell lines obtained from the G418 or GS selection systems in semi-solid CloneMedia (Genetix, New Milton, UK). For each pool of stably transfected cells 12,000 cells were added to 24ml of CloneMedia supplemented with 8mM Glutamax. The mix was plated at 2ml/well (500cells/ml) into 6 well Equiglass cell culture plates (Genetix). Clones were picked using a Clonepix^FL^ robot (Genetix) under white light illumination into 96 well plates containing XP media (Genetix) supplemented with 1.5mg/ml G418 or 50μM MSX.

### Antibody expression analysis by ELISA

Supernatant from the tissue culture flasks was removed and antibody titre within this determined using a mouse IgG ELISA. Nunc 96 well plates were coated with goat anti-mouse Fc antibody by loading with 100μl of a 2μg/ml solution in PBS and incubation overnight at 4°C. Plates were then rinsed twice with wash buffer [PBS, 1% (v/v) Tween 20] and blocked with 200μl/well blocking buffer consisting of PBS, 3% (w/v) BSA and 1% (v/v) Tween 20 for 1 hour at 15–25°C. Plates were washed as before and test samples added to the top row of wells. In addition, a mouse IgG1 standard, MOPC21 (UCB, Slough, UK) (at a concentration of 1μg/ml) was added in duplicate and samples were titrated at 1 in 3 down the plate in conjugation buffer [PBS, 0.5% (w/v) BSA, 0.2% (v/v) Tween20]. After 1 hour incubation the plates were washed again and 100μl/well goat anti-mouse Fc-HRP (Jackson ImmunoResearch, Stratech Scientific Ltd, Newmarket, UK) at a 1:5000 dilution in conjugation buffer was added to all wells and incubated at 15–20°C for 1 hour. Plates were then washed and binding revealed using TMB (Calbiochem, San Diego, CA, USA). Plates were read at 630nm and concentrations calculated using KC4 data analysis software (Biotek, UK).

### Southern blot analysis for diversity and DNA integration

Genomic DNA (gDNA) was extracted from CHO-K1 cells using the Wizard Genomic DNA Isolation Kit (Promega, Southampton, UK) according to the manufacturer’s instructions.

Southern blot hybridisation analysis was conducted using the Digoxigenin (DIG) labelled probe synthesis method. PCR was used to generate DIG labelled hybridisation probes using the Expand High Fidelity PCR system (Roche Diagnostics Ltd, Burgess Hill, UK) in a total volume of 50μl according to the manufacturers’ instructions. The probe generated for the Southern blot hybridisation was a fragment of the ampicillin bacterial selection marker which is capable of annealing to the 3’ end of the linearised antibody expressing vector (Table 1).

Total gDNA (10μg) was digested to completion using *Eco*RI restriction enzyme for 16 hours. Digested DNA, along with DIG labelled DNA Molecular weight marker (Roche Diagnostics Ltd) were resolved by electrophoresis on a 0.8% (w/v) Tris-Acetate-EDTA (TAE) agarose gel. The DIG hybridisation protocol was followed according to the manufacturer’s instructions (Roche Diagnostics Ltd) for depurination, denaturation and neutralisation of the gel, the capillary transfer of the DNA to the membrane and the hybridisation of the probe to the membrane. A hybridisation temperature of 45°C was calculated and applied. After hybridisation the membrane was washed, blocked and the anti-DIG antibody applied according to the manufacturer’s instructions (Roche Diagnostics Ltd). Finally the chemiluminescent alkaline phosphate substrate CSPD was added and the membrane exposed to X-ray film (Kodak, Hemel Hempstead, UK) for at least 30 minutes and the film developed using a compact X4 automatic X-ray film processor (Xograph Healthcare Ltd, Tetbury, UK).

### Heavy chain and Light chain mRNA quantification

Total RNA was extracted from CHO-K1 cells using the RNeasy plus kit (Qiagen Ltd, Manchester, UK) according to the manufacturer’s instructions. RNA was converted into cDNA using a High Capacity cDNA RT kit (Life Technologies Ltd, Paisley, UK) according to the manufacturer’s protocol. Quantitative Real Time PCR assays were performed on 2ul of cDNA in 384-well optical plates on an ABI Prism 7900HT Sequence Detection system (Life Technologies Ltd, Paisley, UK). Each sample was assayed in triplicate. A master mix was prepared which contained for each 10μl TaqMan reaction, 2μl DNA template, 2.83μl dH2O, 0.17μl 60x TaqMan probe (consisting of forward and reverse primers, each with 1x concentration of 900nM and a 6-FAM dye-labelled TaqMan probe, 1x concentration, 250nM) and 5μl 2x Quantitect mix (containing HotStar DNA polymerase, Quantitect probe PCR buffer, dNTP mix, ROX passive reference dye, 8mM MgCl2) (Qiagen Ltd, Manchester, UK). The samples were cycled under the following conditions: 50°C for 2 minutes, 95°C for 10 minutes, and 40 cycles of 95°C for 15 seconds, 60°C for 1 minute. The results were analysed using the SDS 2.3 software for absolute quantification calculations or RQ manager software for relative quantity determinations (Life Technologies Ltd, Paisley, UK). HC, LC and GAPDH probes (Table 1) were used and the amount of HC and LC was expressed relative to the level of GAPDH that was detected.

### Fluorescence *In Situ* Hybridisation

For the fluorescence *in situ* hybridisation (FISH) the A2UCOE antibody expression vector was used as the probe. Nick translation (NT) was used to fragment the vector DNA into <500bp a reaction mixture containing 1x NT buffer, 10mM β-mercaptoethanol, 0.5mM each of dATP, dCTP, 0.1mM dTTP, and 1mM biotin labelled dUTP (Roche Diagnostics Ltd.), 5ng DNase, 10U *E*.*coli* DNA polymerase I (New England Biolabs, Hitchin, UK) and 1μg template DNA in a final reaction volume of 50μl was used. The reaction was incubated at 16°C for 90 minutes and quenched by addition of 5μl of 0.5M EDTA, pH 8. The metaphase chromosome spreads from the CHO-K1 cells were prepared according to the method cited in Lattenmayer, 2006 [36].

### Determining the level of DNA methylation by bisulphite conversion and sequencing

The complete conversion of unmethylated cytosines to uracils in 1μg gDNA was carried out using the EpiTect Bisulfite kit (Qiagen Ltd) according to the manufacturer’s instructions. Following the bisulphite conversion and purification of the DNA, a first round of PCR reactions was conducted using Taq polymerase. Oligonucletoides were designed to specifically amplify the hCMV-MIE promoter driving either the HC (700bp) or LC (660bp) (Table 1). The reaction mix contained 8μl of bisulphite converted DNA, 10pmoles of forward and reverse primers, 0.2mM dNTP mix, 5μl 10X PCR buffer (100mM Tris-HCl, 15mM MgCl_2_, 500mM KCl, pH 8.3), 1.25U Taq polymerase and sterile dH_2_O to a final volume of 50 μl. Reactions were PCR cycled under the following conditions: 94°C for 2 minutes, 94°C for 30 seconds, 55°C for 30 seconds, 64°C for 45 seconds and repeated for 40 cycles and 64°C for 7 minutes. An 8μl aliquot of the PCR reaction from the first round of reaction was used in a subsequent nested PCR reaction. Reactions were set up and cycled under the same conditions as the first round PCR.

## Results

### Expression studies to determine the preferred element and its optimal configuration

**Fig 1:**
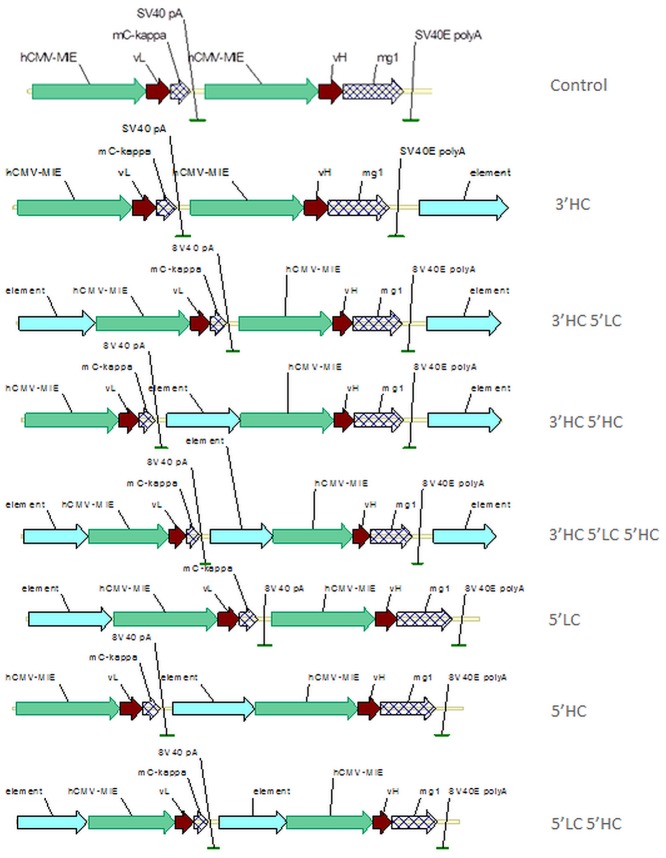
Configuration of immunoglobulin heavy chain (HC) and light chain (LC) antibody sequences. Diagrams illustrating the configuration of the immunoglobulin heavy chain (HC) and light chain (LC) antibody gene sequences relative to the location of the chromatin function modifying elements inserted within the vector. Top line: control plasmid vector showing in order hCMV promoter (green arrow)-LC gene cassette (tandem brown and hashed arrows), hCMV-HC gene unit. Sites of insertion of the chromatin function modifying elements are denoted as blue arrows.

**Fig 2:**
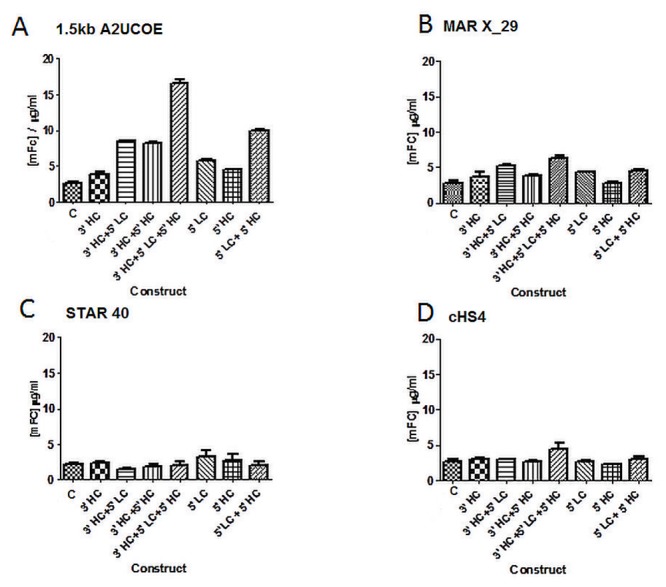
A2UCOE-augmented vectors provide the highest elevation in antibody production in stably transfected cell pools. The control and chromatin element containing vectors as depicted in Fig 1, were introduced into CHO-K1 cells and stably transfected cell pools selected with G418. Cells were then seeded at 3x10^5^ cells/ml and antibody production measured after 10 days using a mouse IgG ELISA. Each data point represents mean +/- SD (n = 2). (A) 1.5kb A2UCOE, (B) MAR X_S29, (C) STAR40, (D) cHS4.

We began by establishing the optimal location and combination for the individual A2UCOE, MAR X_S29, STAR40 and cHS4 chromatin function modifying elements for maximum levels and stability of expression. A total of seven constructs for each were generated (Fig 1) with 1, 2 or 3 copies of these elements located at different positions relative to the HC and LC antibody gene cassettes within the expression vector. The linearised vectors were then transfected into CHO-K1 cells and stable transfectants under G418 selection were obtained. Recovered pools were analysed for antibody expression (Fig 2). Incorporation of two A2UCOE elements (5’ LC 5’ HC) resulted in a 3-fold increase in expression, and the presence of three elements (3’ HC 5’ LC 5’ HC) gave a 6.5-fold increase over those obtained with the control expression vector (Fig 2A). The remaining three MAR X_S29, STAR40 and cHS4 elements in tandem, increased expression levels to a much lower extent, with at best a 2-fold increase observed in the case of MAR X_S29 (3’ HC 5’ LC 5’ HC) (Fig 2B–2D).

**Fig 3:**
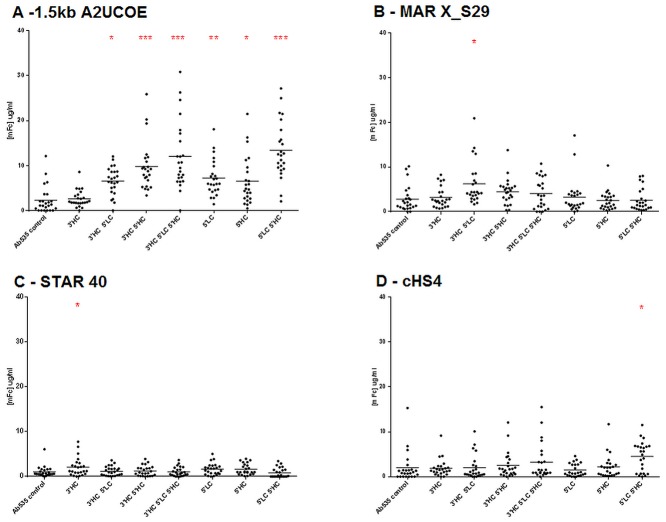
A2UCOE provides higher mean antibody expression levels in stably transfected CHO-K1 cells clones. From a total of 192 initially selected clones harbouring either expression vectors containing the control or chromatin elements 24, clones with the highest expression levels from each set were seeded into 24 well tissue culture plates and antibody production measured 10 days later. Graphs show the range of expression levels with the mean expression level highlighted by horizontal bar for each cell line for the four elements. A multi-comparison 1-way ANOVA Dunnetts analysis was performed with 95% CI * p<0.05, ** p<0.01 and *** p<0.001. (A) 1.5kb A2UCOE, (B) MAR X_S29, (C) cHS4, (D) STAR 40.

In order to ascertain the frequency of expressing clones within each stably transfected pool, clonal cell lines were isolated from the pools using the white light ClonePix selection method. A total of 192 clones for each construct were picked and analysed for antibody expression after 7 days of culture (data not shown). The 24 highest expressing clones from each construct were then cultured for a further 10 days in 24 well tissue culture plates after which expression levels were re-analysed. Increased expression was observed for all clones containing the A2UCOE-element with the exception of the 3’ HC construct which was similar to the control (Fig 3A). Clones with the highest antibody titre were isolated from the cells transfected with the A2UCOE 3’ HC 5’ LC 5’ HC construct with these showing expression levels 2.5-fold higher than the best expressing clone harbouring the control vector. The mean expression level for all 24 clones analysed for each construct showed that the highest single increase observed was with a clonal cell line generated using the A2UCOE 3’HC 5’ LC 5’ HC vector (6.75-fold increase).

For clones derived from vectors containing the MAR X_S29 element, the highest expression levels were observed when placed at the 3’ HC 5’ LC locations (Fig 3B), with the average expression level from these clones 2.2-fold higher than the average from the control construct (Fig 3B). Analysis of the STAR40-containing clones showed that irrespective of the position or number of this element present within the test vector, the average expression levels of the cell lines from each construct were similar to that of the control (Fig 3C). Again these results were consistent with the results observed previously for the stably transfected pools (Fig 2). The highest peak expression level observed for the cHS4 tandem element was in the cell lines generated using the 3’ HC 5’ LC 5’ HC construct (Fig 3D), but the highest average expression level observed was with the 5’ LC 5’ HC vector (Fig 3D).

**Fig 4:**
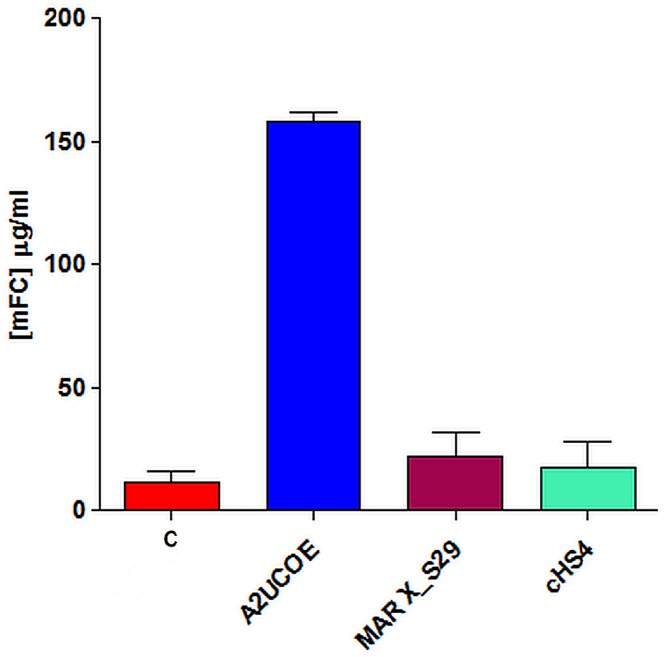
A2UCOE-augmented vectors provide higher antibody expression in CHO-K1 cells following GS selection. The A2UCOE (3’ HC 5’ LC 5’ HC), MAR X_S29 (3’ HC 5’LC) and cHS4 (5’LC 5’HC) constructs that gave the highest yields following G418 selection (Fig 2) were stably transfected and selected using the GS system. Cells were then seeded at 3x10^5^ cells/ml and expression measured after 10 days using a mouse IgG ELISA. Each data point represents mean +/- SD (n = 3) of three independent transfections.

In order to further clarify the capabilities of the chromatin elements under analysis, the optimal arrangement for each was further examined using an alternative selection system in case this influences the process of cell line generation. For this study the vector configurations that gave the highest mean expression levels in the G418 selection experiments (Figs 2 and 3) namely 3’ HC 5’ LC 5’ HC (A2UCOE) 3’ HC 5’LC (MAR X_S29) and 5’LC 5’HC (cHS4)) were investigated. In light of the poor STAR40 results in G418 selection further analysis of constructs containing this element were not undertaken. The selected vectors as well as the control construct were stably transfected into CHO-K1 cells under Glutamine Synthetase (GS) selection and expression analysis was carried out following 10 days of growth once cells recovered and cell viability was >90%. Similar to the cell lines generated under G418 selection, the highest expression levels were observed from GS stable pools harbouring the A2UCOE construct where a 15-fold increase in expression was achieved over the control (Fig 4). The MAR X_S29 and cHS4 tandem elements using the GS selection system again showed only a modest increase in expression over the control with a 2- and 1.5-fold increase respectively (Fig 4). In addition, the greater selection stringency of the GS system resulted in a higher level of expression with the 3’ HC 5’ LC 5’ HC A2UCOE vector compared to the same construct following G418 selection (Fig 2).

**Fig 5:**
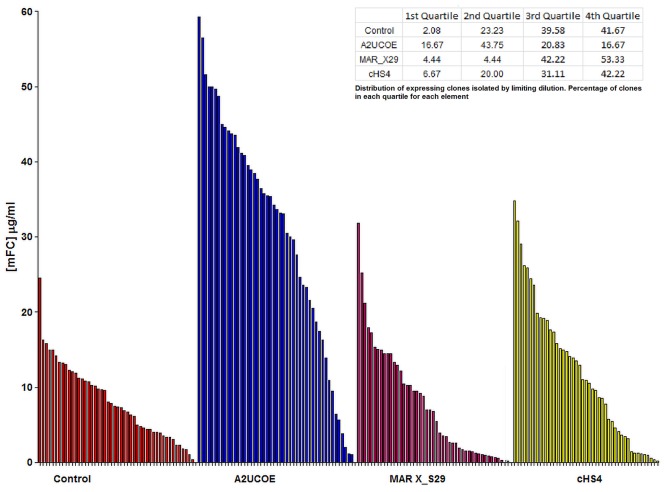
Comparative analysis of antibody yields from clones selected using the GS selection system. Following an initial isolation of 192 clones from the stably transfected CHO-K1GS selected pools containing either the control or 1.5kb A2UCOE (3’ HC 5’ LC 5’ HC), MAR X_S29 (3’ HC 5’LC) and cHS4 (5’LC 5’HC) constructs (Fig 4), the 48 highest expressing clones were seeded and expanded in 24 well tissue culture plates and grown for 10 days. Supernatant was then assayed for antibody production using a mouse IgG ELISA. As can be seen clones containing the 1.5kb A2UCOE construct provide a highest overall level of antibody production. The percentage distribution of clones in each quartile is shown for each element.

A total of 192 clonal cell lines were isolated from these pools using the white light ClonePix method as in the case of the G418 selected clones. Following initial antibody titre analysis at the 96 well plate stage (data not shown) the highest 48 expressing clones from each construct were transferred to 24 well tissue culture plates and supernatants re-analysed for expression following a further 10 days of culture. All A2UCOE-containing clones consistently performed well compared to the control and the other elements analysed with clones isolated from the A2UCOE (3’ HC 5’ LC 5’ HC) demonstrating higher antibody expression levels than those isolated from the stable pool harbouring the control construct (Fig 5). The expression levels for the MAR X_S29 (3’ HC 5’LC) and cHS4 (5’LC 5’HC) tandem clones were similar to the control clones. The cell lines derived from the A2UCOE (3’ HC 5’ LC 5’ HC) transfection consistently showed increased expression compared to the control and were thus taken forward for further molecular genetic characterisation.

### Molecular genetic characterisation of cell lines stably transfected with A2UCOE constructs

The expression level and stability of a cell line can be dependent upon both transgene copy number and site of integration. In order to identify the molecular differences or similarities between clones and understand how the A2UCOE element might function to provide the observed elevated and stable levels of antibody expression within CHO cells, a panel of the stably derived clonal cell lines were analysed using Southern blotting. Southern blot analysis was conducted with ten A2UCOE (3’ HC 5’ LC 5’ HC) and ten control vector CHO-K1 cell clones isolated from the stably transfected pools to determine the diversity and transgene integration pattern of the clones.

**Fig 6:**
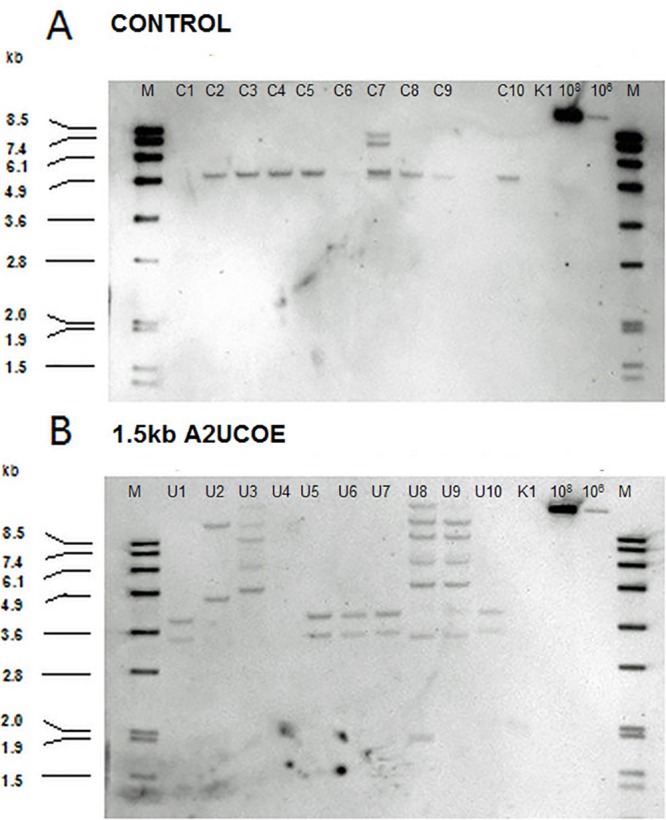
Southern blot analysis of control and A2UCOE vector-containing clones. Total genomic DNA from CHO-K1 clones harbouring stably transfected 1.5kb A2UCOE (3’ HC 5’ LC 5’ HC) and control vectors were digested with *Eco*RI restriction enzyme and subjected to Southern blot analysis with hybridisation using a DIG labelled probe spanning the ampicillin resistance gene on the plasmid vector backbone. (A) C1–C10 = control vector clones. (B) U1–U10 = A2UCOE (3’ HC 5’ LC 5’ HC) vector clones. M = DIG labelled DNA size (kb) ladder. K1 = untransfected CHO-K1 gDNA. 10^8^ = 1 x 10^8^ plasmid copies. 10^6^ = 1 x 10^6^ plasmid copies.

A hybridisation band at ~5kb was observed for seven out of ten control clones analysed (Fig 6A, lanes C2–C5 and C8–C10). This 5kb band equates to the insertion of a single plasmid copy into the genome at the same site. Based upon the Southern blot analysis these clones were grouped into Family A. Clone C7 contains two additional bands at ~7.4 and 8kb (designated Family B). The 7.4kb band is consistent with two copies of the plasmid being inserted in a head-to-tail tandem array. The 8kb band suggested that an additional copy of the plasmid had been integrated at another location within the genome, or has been integrated in a different arrangement. No bands were obtained for clones C1 and C6 indicating that the integrated transgenes lacked plasmid DNA corresponding to the probe region. This can occur by deletion of the 3’ end of the plasmid at or prior to the time of integration (Family C) (Fig 6A and Table 2).

**Table 2:** Summary of cell line families harbouring the HC and LC antibody control (C) and A2UCOE (U) vectors as determined by Southern blot Analysis.

Cell line	Southern blot copy number	Percentage (%) Expression remaining at 120 generations	Percentage (%) HC Methylation		Percentage (%) LC Methylation	
			Generation	Generation	Generation	Generation
			0	120	0	120
**CONTROL**						
**Family A**						
C2	1	<5				
C3	1	<10				
C4	1	<10	4	25	3	30
C5	1	<10				
C8	1	<35	3	10	2	5
C9	1	<5				
C10	1	<10				
**Family B**						
C7	3	<25	13	28	7	22
**Family C**						
C1	0	0				
C6	0	0				
**A2UCOE**						
**Family D**						
U1	2	<85				
U5	2	<70				
U6	2	60				
U7	2	75	1	1	1	2
U10	2	80				
**Family E**						
U2	2	<60	1	9	1	3
**Family F**						
U3	5	<45				
U8	7	<30	12	24	2	15
U9	6	<65				
**Family G**						
U4	0	<25				

The copy number, percentage of antibody expression and HC and LC methylation level after 120 generation.

Of the ten A2UCOE transgene-containing clones analysed U1, U5–U7 and U10 showed two bands, which migrated above and below the 3.6kb size marker fragment (Fig 6B; designated as Family D). These bands could correspond to two single copies integrated at different points within the genome. Clone U2 also showed two bands that were at <4.9kb and >8.5kb (Family E) with the latter possibly corresponding to the integration of plasmids in a head-to-tail tandem array (expected size of 10.9kb). The remaining clones (U3, U8 and U9) showed similar banding patterns (Family F). Seven bands were observed for clone U8 and fewer with U3 and U9. However, the majority of band sizes were the same indicating that these three clones may be daughter progeny from one originally transfected cell. Interestingly, no bands were obtained with clone U4, which could be due to deletion at the 3’ end of the construct (Family G) (Fig 6B). Overall the Southern blot analysis suggested that the clones derived from the control stable pools fell into 3 distinct families (A-C) and the A2UCOE clones into four families (D-G) (Table 2).

**Fig 7:**
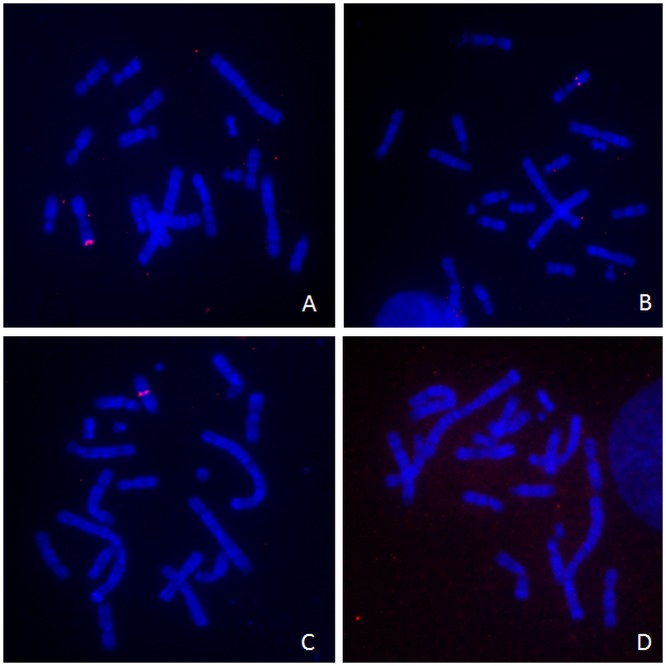
Chromosomal integration sites by Fluorescence In Situ Hybridisation. Biotinylated probes were generated using the 1.5kb A2UCOE plasmid and hybridised to metaphase spreads for the three 1.5kb A2UCOE (3’ HC 5’ LC 5’ HC) construct-containing CHO-K1 clones (Fig 6). Sites of probe hybridisation were detected with streptavidin conjugated to Cy3 and integration sites are revealed as pink dots. Chromosomes were highlighted by counterstaining with DAPI. Panel A: clone U2; Panel B: clone U8; Panel C: clone U9. Panel D: untransfected CHO-K1 cells.

Further information concerning chromosomal sites of A2UCOE construct transgene integration was obtained by conducted fluorescence in situ hybridisation (FISH) analysis of metaphase spreads of clones U2 (Family E), U7 (Family D) and U8 (Family F). A total of ten metaphase FISH spreads were analysed for each of these clones. Untransfected CHO-K1 cells were used as a negative control and the lack of signal seen with these cells indicated that the probe was transgene DNA-specific (Fig 7D). The results show that for all three A2UCOE clones there is one region of integration (Fig 7A–7C). In two of the clones, U2 and U8, it appeared that the transgene plasmid DNA had integrated into heterochromatic regions; the telomere for U2 and the centromere for clone U8 (Fig 7A–7C). As telomeres and centromeres are regions of transcriptionally non-permissive heterochromatin, little or no expression would be expected to take place following transgene integration into these areas.

### Assessing the stability of expression in A2UCOE cell lines

We next assessed whether the A2UCOE was able to positively affect the stability of HC and LC transgene mRNA expression levels and negate CMV promoter methylation-mediated silencing as has been seen in other systems [13, 17]. Clones were cultured in 6 well plates and continuously passaged for 120 generations and with antibody productivity assessed at 0, 40, 80 and 120 generations, with 0 generations being the point at which MSX was removed from the culture medium, at approximately four weeks after single colonies were isolated.

**Fig 8:**
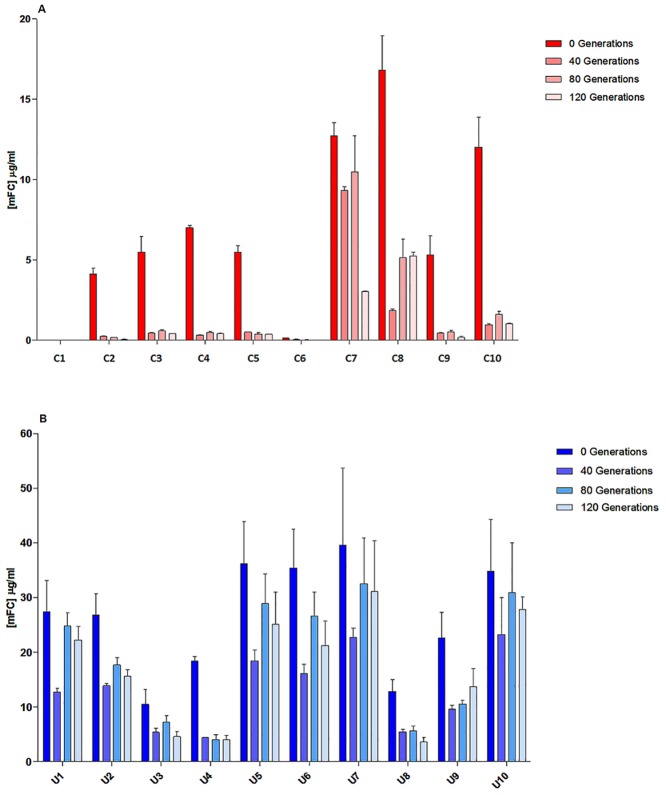
Stability of antibody expression from clones harbouring control and A2UCOE (3’ HC 5’ LC 5’ HC) vectors cultured in the absence of drug selective pressure. Stably transfected CHO-K1 clones containing the control and 1.5kb A2UCOE (3’ HC 5’ LC 5’ HC) vectors (Fig 6) were continuously cultured for 120 generations without MSX selective pressure. Overgrow cultures of 10 day duration were set up at 0, 40, 80 and 120 generations. Supernatant was then assayed by mFc ELISA to establish antibody expression levels. The percentage of expression remaining after 120 generations is also shown. (A) control vector (B) 1.5kb A2UCOE construct. Each data point represents mean +/- SD of triplicate determinations.

Expression analysis showed that clones containing the control construct from Family A were unstable, with their expression levels decreasing to <10% of the starting (0 generation) level by 40 generations (Fig 8A). The exception was clone C8, which showed reduced expression to 35% of its original level by 120 generations (Fig 8A). Clone C7 (Family B) maintained its expression level for ~80 generations and then a marked (~4-fold) reduction was observed in the last 40 generations.

The A2UCOE clone (U7) analysed from Family D had the highest starting levels of expression (28–40μg/mL) at 0 generations and showed only a 25% reduction in expression after 120 generations (Fig 8B). Family F (clone U8), which harboured >5 transgene copies per cell as determined by Southern blot analysis (Fig 6B), had lower starting expression levels (10–22μg/mL) and showed a reduction equivalent to Family D but with their overall expression level lower after 120 generations (Fig 8B). Clone U2 from Family E showed a 40% decrease in expression by 40 generations and then no further reduction was observed (Fig 8B).

**Fig 9:**
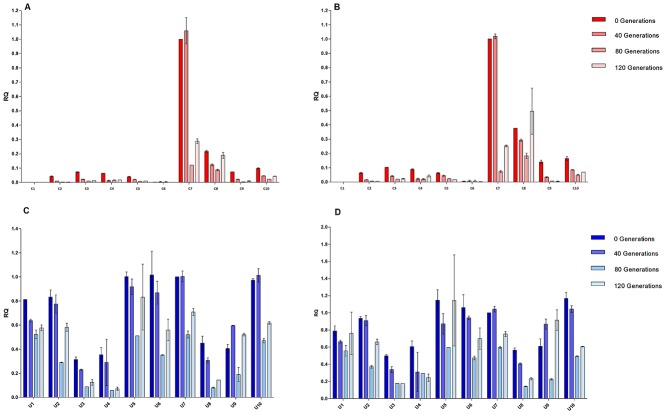
Relative mRNA levels parallel antibody production during long term culture in the absence of selection pressure. Relative HC and LC mRNA levels were quantified at 0, 40, 80 and 120 generations using RT-Q-PCR. RQ values were calculated using the 2^-ΔΔ CT^ method. Input RNA quantity was normalised using GAPDH as an internal control. Each data point represents mean +/- SD of two independent RT-QPCR assays.

In order to determine if the reduced expression level of the control and A2UCOE clones over 120 generations was due to a reduction in HC and LC gene transcription, mRNA levels were quantified using reverse transcriptase real-time quantitative-PCR (RT-Q-PCR). Total RNA was extracted from the 10 control and 10 A2UCOE clones (Fig 6) at 0, 40, 80 and 120 generations of continuous culture. The results (Fig 9) show that the HC and LC mRNA profiles correlate closely with antibody production profiles (Fig 8) over the period of 120 generations for both the control (Fig 9A and 9B) and A2UCOE (Fig 9C and 9D) constructs. Where a decrease in antibody expression was observed, this was accompanied by a decrease in mRNA levels. Furthermore, Southern blot analysis showed that the transgene banding pattern was largely similar at 120 generations as that seen at generation 0 (data not shown).

**Fig 10:**
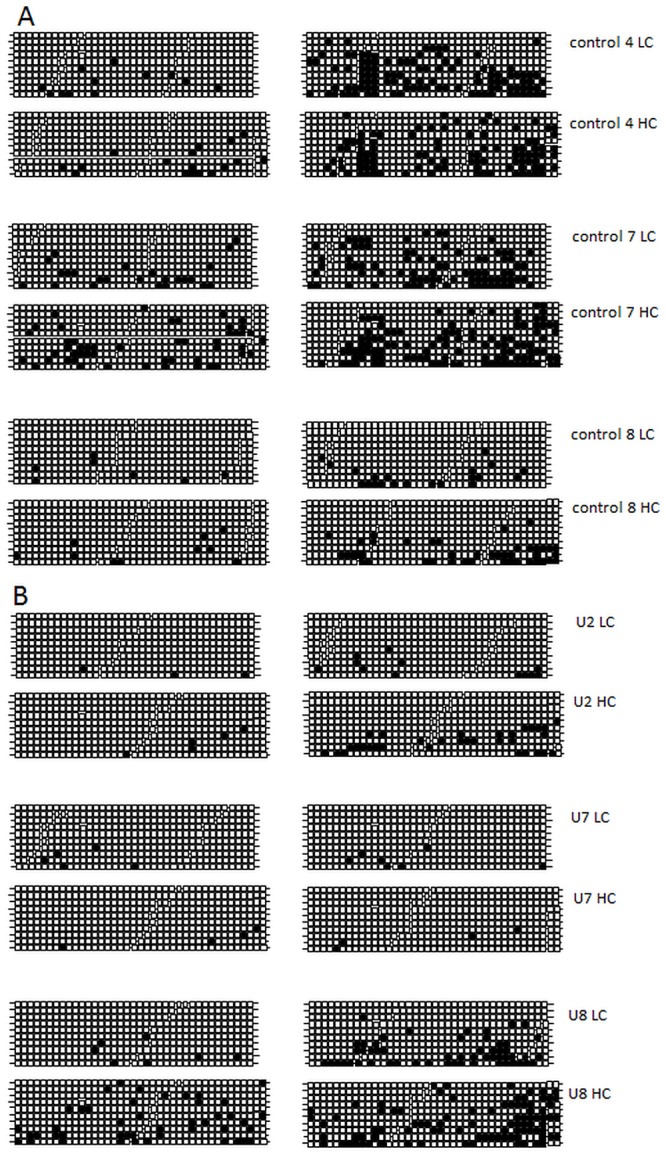
DNA methylation status of CpG dinucleotides within the CMV promoter regions driving HC and LC expression in (A) control (B) A2UCOE (3’ HC 5’ LC 5’ HC) clones. Bisulphite conversion and DNA sequencing was performed to establish the number of methylated cytosine residues in CpG dinucleotides within the hCMV-MIE promoters. Following bisulphite conversion, the hCMV-MIE regions were amplified by PCR and products cloned and sequenced. Each line represents the sequence of an individual PCR amplified plasmid clone from the hCMV-MIE region. Each square on a given line represents the position of a CpG dinucleotide within the amplified hCMV-MIE region. White squares, unmethylated CpG dinucleotide; black squares, methylated CpG dinucleotide.

In order to investigate if the decline in expression observed over time was a result of epigenetic regulation via DNA methylation, bisulphite conversion followed by sequencing of the CMV promoter regions driving the HC and LC transgenes was investigated for clones C4 (Family A), C7 (Family C) and C8 (Family A) and U2 (Family E), U7 (Family D) and U8 (Family F). Specific PCR amplification distinguishing the HC and LC hCMV-MIE promoters following bisulphite conversion was achieved by using oligonucleotide primers corresponding to a fragment of DNA encompassing the 3’ region of the promoter and the 5’ region of the vL (660bp) or the vH (700bp) genes. This allowed a total of 37 and 39 CpG dinucleotides to be analysed in the LC and HC CMV promoter regions respectively. The control clones all showed an increase in the percentage of HC and LC CMV promoter methylation over 120 generations (Fig 10a). Clone C8 (family A) had the lowest levels of CMV promoter methylation after 120 generations (HC-10%, LC-5%), which correlated with the lowest decrease in expression at this time point amongst the control group. The A2UCOE clones U7 (family D) and U2 (family E), which both contained 2 plasmid copies as determined by Southern blot analysis, showed no substantial increase in CMV promoter methylation (Fig 10b). For clone U7 the level of CMV promoter methylation remained unchanged for 120 generations, which corresponded to only a 25% reduction in antibody expression over this time period. Clone U8 (family F), which contained >5 transgene copies, showed a high level of CMV promoter methylation at 0 generations and this increased further at 120 generations compared to the other clones. This clone also displayed a reduction in HC and LC mRNA level and a reduction in antibody expression of ~70% at this time point (Fig 10b).

## Discussion

Whilst various elements with a capability to either modify or maintain chromatin architecture in a transcriptionally permissive state have been described, in particular UCOE, MAR, STAR and the cHS4 insulator, there is limited information on their use for the generation of stable antibody producing cell lines. This study presents the first direct comparison of UCOE, MAR, STAR and the cHS4 insulator elements to provide high, reproducible and stable levels of antibody expression within the same vector system and within an industrially relevant CHO cell line.

There are many reports and reviews on the use of chromatin modifying elements to augment function of reporter genes such as GFP and luciferase [7, 11, 13–14, 23–25, 34] but far less data exists on the potential benefits of these elements for the generation of industrially relevant stable antibody expression systems. In addition, the experimental parameters between the reports that have assessed antibody production have also differed widely [10]. Our study utilised vectors which contained both HC and LC gene cassettes on the same plasmid and the chromatin modifying elements assessed in a variety of positions relative to the promoters driving expression.

Following transfection and selection of stable cell pools, our data showed that the UCOE, MAR, STAR and cHS4 elements displayed a marked difference in efficacy. The A2UCOE proved to be most potent in providing both elevated and stable antibody production when compared to the other elements. Our data also revealed that the position of the chromatin modifying elements within the vector was also crucial for optimal function and higher antibody production.

The 1.5kb core A2UCOE element studied here is derived from the human *HNRPA2B1-CBX3* locus [11, 13] and when incorporated into our HC/LC vector showed the greatest benefit with respect to increasing antibody expression levels (6.75-fold) obtained from stably transfected pools of CHO cells. This increase in expression has also been observed with a larger 4kb A2UCOE in expression of ScFvs in CHO-S cells [37].

We found that the MAR X_29 was capable of increasing antibody titre by up to 2-fold, which is consistent with a study showing a 2.1-fold increase of CAT reporter gene expression [34] but >2-fold lower than EGFP expression in CHOK1 cells [38]. In our study This reduced degree of benefit from MAR X_S29 we observed, may be explained by the observation that only a subset of MAR type elements have activity in CHO cells [39].

In our initial experiments the STAR40 element showed limited activity in CHO cells regardless of either their number or position within the plasmid vector. These observations contrast with the study, which first described the STAR40 element where increased numbers of stably transfected clones and expression levels of secreted alkaline phosphatase (SEAP) in CHO cells were reported [25]. However, subsequent to this initial report STAR elements (STAR7 and STAR67) with a greater efficacy in CHO cells have been described, particularly when used in combination [40–42]. Whether the STAR7 and STAR67 elements match or outperform the efficacy of the 1.5kb A2UCOE is at present unknown.

Use of the cHS4 element in this study resulted in similar expression levels to that of the control with only a small increase in the number of clones observed. Our data also confirms the work of others who have shown that the incorporation of the cHS4 insulator in vectors and transfection in CHO cells had limited activity [34, 40]. The cHS4 element is functionally defined as an insulator and thus can block activity of a distal enhancer on a promoter when placed between the two, and can reduce transgene insertion site position effects by acting as a ‘barrier’ [43]. Therefore, cHS4 may not be able to re-model surrounding chromatin structure compared to UCOEs, which have been shown to ‘open’ chromatin and allow expression even from within heterochromatic regions [11, 13].

In order to understand how the 1.5kb A2UCOE enabled the generation of higher expressing stable pools and clonally derived cells, a panel of clonal cell lines containing the 1.5kb A2UCOE regulated cassettes were genetically characterised. The clonal cell lines derived from the stable pools were isolated once the cell viability within a stable pool was >90%. At this relatively late stage in cell population production, it is possible that a given pool may be dominated by a small subset of clones with more favourable properties especially higher mitotic index. This limited diversity is indeed suggested by the grouping of the resultant clones containing either A2UCOE or control vectors into distinct families with similar copy number and transgene integration patterns. An alternative approach, which avoided the initial generation of transfected cell pools, may have generated a panel of stable clonal cell lines with a larger repertoire of integration events as this method is not biased towards clones with favourable growth properties. Nevertheless, analysis of all the clones within the A2UCOE and control vector families showed that HC and LC transgene copy number was below 10 in both cases. This comparable, low transgene copy number between A2UCOE and control transfected cells is in agreement with previous results, which showed that the A2UCOE does not result in an increase in the number of integrated plasmids [13]. In addition, clones which belonged to the same family exhibited similar antibody expression profiles over 120 generations, with comparable starting levels and loss of expression during the continuous culture period. In most cases the A2UCOE conferred stability compared to the control with the lowest decrease in expression observed with these clones over 120 generations. Furthermore, DNA methylation analysis of the CMV promoters driving the HC and LC genes also showed that the lowest DNA methylation levels were observed in the clones harbouring A2UCOE vectors. Clone U7 showed no increase in methylation and the expression after 120 generations was <80% of the starting level whilst C5 showed ~25% increase in methylation and a consequent reduction to <10% of the original generation 0 level. FISH analysis also suggested that A2UCOE clones U2 and U8 contained transgene integration events within heterochromatin and these two families showed the largest reduction in expression and increase in methylation. However, the reduction in level of expression for both of these clones was mitigated compared to the control. These results indicate that the A2UCOE can inhibit the negative effects of the surrounding heterochromatin at the site of transgene integration as previously observed [11, 13]. The result of the effect of the A2UCOE was not unexpected as recent reports demonstrated that this element can avoid silencing by being immune to DNA methylation and can confer this property on linked heterologous ubiquitous [14–15] and tissue specific [16] promoters in murine embryonic carcinoma P19 [16, 18] induced pluripotent stem [15] and haematopoietic stem [14, 16] cells. In these cells the stability of transgene expression from the A2UCOE within self-inactivating lentiviral vectors (SIN-LVs) was shown to be due to the transgene being resistant to DNA methylation. Epigenetic analysis of the *HNRPA2B1-CBX3* locus has found that a large region of the DNA is free from methylation beyond the boundaries of the CpG island spanning the divergent promoters of these genes, and histone modifications associated with active transcription are present [44]. This has led to the model that the mechanism of UCOE function is due to the reproduction of these active epigenetic marks at the site of transgene integration, which then prevents DNA methylation and heterochromatin formation and thus prevents transcriptional silencing [14].

In summary, this study investigated four different chromatin modifying elements using an industrially relevant antibody production plasmid vector and cell line system. Our results showed that of the four elements tested, the A2UCOE was the most effective in providing reproducible and stable expression relative to the control. The incorporation of the A2UCOE enabled higher expressing stable pools of cells to be generated and the subsequently derived clones to be more stable than those generated with the control vector lacking this element.

## Supporting Information

S1 Fig Sequences of Chromatin function elements. (DOC)
