# Supplementary material for: Chromatin Function Modifying Elements in an Industrial Antibody Production Platform - Comparison of UCOE, MAR, STAR and cHS4 Elements
Source: PLoS One. 2015 Apr 7;10(4):e0120096. doi: 10.1371/journal.pone.0120096 (PMC4388700; doi:10.1371/journal.pone.0120096)
Supplement: S1 Fig — (DOC) [file pone.0120096.s001.doc]

**Chromatin modifying element sequences**

**1.5kb A2UCOE**

GCGGCCGCACGCGTGGCCCTCCGCGCCTACAGCTCAAGCCACATCCGAAGGGGGAGGGAGCCGGGAGCTGCGCGCGGGGCCGCCGGGGGGAGGGGTGGCACCGCCCACGCCGGGCGGCCACGAAGGGCGGGGCAGCGGGCGCGCGCGCGGCGGGGGGAGGGGCCGGCGCCGCGCCCGCTGGGAATTGGGGCCCTAGGGGGAGGGCGGAGGCGCCGACGACCGCGGCACTTACCGTTCGCGGCGTGGCGCCCGGTGGTCCCCAAGGGGAGGGAAGGGGGAGGCGGGGCGAGGACAGTGACCGGAGTCTCCTCAGCGGTGGCTTTTCTGCTTGGCAGCCTCAGCGGCTGGCGCCAAAACCGGACTCCGCCCACTTCCTCGCCCGCCGGTGCGAGGGTGTGGAATCCTCCAGACGCTGGGGGAGGGGGAGTTGGGAGCTTAAAAACTAGTACCCCTTTGGGACCACTTTCAGCAGCGAACTCTCCTGTACACCAGGGGTCAGTTCCACAGACGCGGGCCAGGGGTGGGTCATTGCGGCGTGAACAATAATTTGACTAGAAGTTGATTCGGGTGTTTCCGGAAGGGGCCGAGTCAATCCGCCGAGTTGGGGCACGGAAAACAAAAAGGGAAGGCTACTAAGATTTTTCTGGCGGGGGTTATCATTGGCGTAACTGCAGGGACCACCTCCCGGGTTGAGGGGGCTGGATCTCCAGGCTGCGGATTAAGCCCCTCCCGTCGGCGTTAATTTCAAACTGCGCGACGTTTCTCACCTGCCTTCGCCAAGGCAGGGGCCGGGACCCTATTCCAAGAGGTAGTAACTAGCAGGACTCTAGCCTTCCGCAATTCATTGAGCGCATTTACGGAAGTAACGTCGGGTACTGTCTCTGGCCGCAAGGGTGGGAGGAGTACGCATTTGGCGTAAGGTGGGGCGTAGAGCCTTCCCGCCATTGGCGGCGGATAGGGCGTTTACGCGACGGCCTGACGTAGCGGAAGACGCCTTAGTGGGGGGGAAGGTTCTAGAAAAGCGGCGGCAGCGGCTCTAGCGGCAGTAGCAGCAGCGCCGGGTCCCGTGCGGAGGTGCTCCTCGCAGAGTTGTTTCTCCAGCAGCGGCAGTTCTCACTACAGCGCCAGGACGAGTCCGGTTCGTGTTCGTCCGCGGAGATCTCTCTCATCTCGCTCGGCTGCGGGAAATCGGGCTGAAGCGACTGAGTCCGCGATGGAGGTAACGGGTTTGAAATCAATGAGTTATTGAAAAGGGCATGGCGAGGCCGTTGGCGCCTCAGTGGAAGTCGGCCAGCCGCCTCCGTGGGAGAGAGGCAGGAAATCGGACCAATTCAGTAGCAGTGGGGCTTAAGGTTTATGAACGGGGTCTTGAGCGGAGGCCTGAGCGTACAAACAGCTTCCCCACCCTCAGCCTCCCGGCGCCATTTCCCTTCACTGGGGGTGGGGGATGGGGAGCTTTCACATGGCGGACGCTGCCCCGCTGGGGTGAAAGTGGGGCGCGGAGGCGGGACTTCTTATTCCCTTTCTAAAGCACGCTGCTTCGGGGGCCACGGCGTCTCCTCGGACGGCCGGGCGCGCC

**MAR X_S29**

GCGGCCGCACGCGTCTCGAGGATCCCTTTATAAAACCACAATATAATGGAGTGCTATAATTTCAAACAGTGTTTGGTCTGCTGGCAGAGTGGTCATTCTAACAGCAGTCACAGTAGAGTAGAAATAAGACTGCAGTATATCTAAGGCAAAAAGCTGAGGTTTCAGGAGCTTGAAGGTAAAGAGGAAGAAAGAAATGGGAATGGGAATTGGAAAGACAAATATCGTTAAGAGAAAATTGCTTTTAGGAGAGGGGAAAGAATCTATGTGTACTTAAGACTATGGAATCAATCCCATTTAAGCTGGGAAACTAGTTTCATATATAACTAATAAATTTTATTTACAGAATATCTATTTACCTGATCTAGGCTTCAAGCCAAAGGGACTGTGTGAAAAACCATCAGTTCTGTCATATTCCTAAAAAAAAATTAAAAAGTTAAAAATAAATAAATAATAAAACTTCTTTTCTTTCAAAATAATCAAGGTGCTTATTCACATCCATTCCAATTTGGGGAAATACTTATTTTCCTATGATTAGTGAAGAGAAAAGTAACTTGCATTTCAATTCAAGTTGATACATGTCACTTTTAAGAGGTCAACTAATATTTGCTAGTTGAGCTAACCATATAGGCTTTAAATACTTTCATAGTAGAAAGAAAATGAAAATCATTAGTGAACTGTATAAAATAGATCATACTTTTTGAAAGAATCAGACTGAAGTTTCCGAAAAAAAGAAGTAAGCTTCAATGAAAAGGTAAGTGAATTTAGCATTTACTCAGCATCTACTATGGACTTAACACCTAACAGTAGATAATCTGAAGGCAAACATATTTGTATAGGGACTGCAGAATGATAGATGATAAATATCATCTCTTCTATTTGAATGAATATTTTTTCAAATCTTTCACACACAGTGGTTTGCTATGGAAAGATTTGTAGTACATTAAACAAATCTGAAGATGGAGTTAGAAAGCTTAGGCTATGTTTTGAGCACAACATATAATTTCTCTGTGATTGTTTCTTCATCTTTCAAATGAGGTTACTGTGAAGATTAAATGAGATAACTAAATGATGATAAAATAATGTAATCTTAGCAGCACCTTATTTAATCTGTGCAACAACTCTGTGAAGTGAGTAGGGCTCAGCTTCAGTCACTTCTCTGCCATTTATTAACTAAGATAGTTTGGAAAGTTACCCATCTCTTCAGCTGTAAAATGATGAGGATCATACCTATTTTATGGGGCTGCTTTTAGGTACAAATATACAGGCAAGCACTTTGTTAATACTAAAGCATTACACCAATTAGTTTTACTCTTTTCCATTCACACATGAAATTAATGTAATCAGAATTCTGTAGATTACCTAAATCTTCTGTTAACACGTGATATGCAGTTCAGGTTAAATGTCAGTTGAGTTACCAAAGCACATACATACTCACCACCCTATCCAAATCTACAAGCCTCCCAGTTTGTCTTCACTATTTTGGTTAAATTAATATGAATTCCTAGATGAAAATTTCACTGATCCAAATGAAATAAAAAATATATTACAAAACTCACACCTGTAATCTCAACATTTTGGGAGGCCAAGGCAGGTAGATCACTTGAGGCCAGGAGTTCAAGACCAGCCTGATCAACATGGTGAAACCCTGTCTCTACTAAAAATACAAAAATTAGCCAGGTGTGGTGGCATGTGCCTGTAGTCCTACCTACTCGGGAGGCTGAGGCACAAGAATCGCTTGAATGTGGGAGGTGGAGGTTGCAGTGACCTGAGATCGTGCCACTGCACTCCAGCCTAGGCAACAGAGTGAGATCATGTGTCATATATATATATATATATATATATATATATATATATATATACACACACACACACATATATATATACACATATATATACGTATATATATATATGTATATATATACATATATATACATATATATATATACGTATATATATACGTATATATATATCAATGTAAATTATTTGGGAAATTTGGTATGAATAGTCTTCCCTGTGAACACAGATCATAAAATCATATATCAAGCAGACAAATAAGTAGTAGTCACTTATATGCTTATACTTGTAACTTAAAGTAAAAGAATTACAAAAGCATATGACAAAGACTAATTTTAAGATATCCTAATTTAAATTGTTTTCTAAAAGTGTGTATACCATTTTACCTATCATATGAATAATTTAGAAACATGTTTATAAAATTAATGTCCAAATCCATTCAAAAGTTTTGTAATGCAGATCACCCACAACAACAAAGAATCCTAGCCTATTAAAAAAGCAACACCACCTACATATAATGAAATATTAGCAGCATCTATGTAACCAAAGTTACACAGTGAATTTGGGCCATCCAACACTTTGAGCAAAGTGTTGAATTCATCAAATGAATGTGTAATCATTTACTTACTAATGCCAATACACTTTAAGGTAATCTTAAGTAGAAGAGATAGAGTTTAGAATTTTTTAAATTTATCTCTTGTTGTAAAGCAATAGACTTGAATAAATAAATTAGAAGAATCAGTCATTCAAGCCACCAGAGTATTTGATCGAGATTTCACAAACTCTAACTTTCTGATACCCATTCTCCCAAAAACGTGTAACCTCCTGTCGATAGGAACAACCCACTGCAGGGATGTTTCTCGTGGAAAAAGGAAATTTCTTTTGCATTGGTTTCAGACCTAACTGGTTACAAGAAAAACCAAAGGCCATTGCACAATGCTGAAGTACTTTTTTCAAATTTAAAATTTGAAAGTTGTTCTTAAAATCTATCATTTATTTTAAAATACGGATGAATGAGAAAGCATAGATTTGATAAAGTGAATTCTTTTCTGCAATCTACAGACACTTCCAAAAATCACTACAGACACTACAGACACTACAGAAAATCATAAATAAACAAGTGCTAGTATCAATATTTTTACCAAAAAATGGCATTCTTAGAATTTTTTATAGGCTAGAAGGTTTGTACAAACTAATCTGCCACGGATTTTAAAATATGAGTGAATAAATTATATTGCAAAAAAAATCAGGTTACAGAGAACTGGCAAGGAAGACTCTTATGTAAAACACAGAAAACATACAAAACGTATTTTTAAGACAAATAAAAACAGAACTTGTACCTCAGATGATACTGGAGATTGTGTTGACATATTAGCATTATCACTGTCTTGCTAAAACATAAAAATAAAAAGATGGAAGATGAAATTACAATACAAATGATGATTTAAACATATAAAAGGAAAATAAAAATTGTTCTGACCAACTACTAAAGGAAGACCTACTAAAGATATGCCATCCAGCACATTGCCACTCTACATGTGGTCTGTAAACCAGCAGCATAGGGATCCATCGGCCGGGCGCGCCGTCGAC

**STAR 40**

GATCAAGAAAGCACTCCGGGCTCCAGAAGGAGCCTTCCAGGCCAGCTTTGAGCATAAGCTCTGATGAGCAGTGAGTGTCTTGAGTAGTGTTCAGGGCAGCATGTTACCATTCATGCTTGACTTCTAGCCAGTGTGACGAGAGGCTGGAGTCAGGTCTCTAGAGAGTTGAGCAGCTCCAGCCTTAGATCTCCCAGTCTTATGCGGTGTGCCCATTCGCTTTGTGTCTGCAGTCCCCTGGCCACACCCAGTAACAGTTCTGGGATCTATGGGAGTAGCTTCCTTAGTGAGCTTTCCCTTCAAATACTTTGCAACCAGGTAGAGAAGTTTGGAGTGAAGGTTTTGTTCTTCGTTTCTTCACAATATGGATATGCATCTTCTTTTGAAAATGTTAAAGTAAATTACCTCTCTTTTCAGATACTGTCTTCATGCGAACTTGGTATCCTGTTTCCATCCCAGCCTTCTATAACCCAGTAACATCTTTTTTGAAACCAGTGGGTGAGAAAGACACCTGGTCAGGAACGCGGACCACAGGACAACTCAGGCTCACCCACGGCATCAGACTAAAGGCAAACAAGGACTCTGTATAAAGTACCGGTGGCATGTGTATTAGTGGAGATGCAGCCTGTGCTCTGCAGACAGGGAGTCACACAGACACTTTTCTATAATTTCTTAAGTGCTTTGAATGTTCAAGTAGAAAGTCTAACATTAAATTTGATTGAACAATTGTATATTCATGGAATATTTTGGAACGGAATACCAAAAAATGGCAATAGTGGTTCTTTCTGGATGGAAGACAAACTTTTCTTCTTTAAAATAAATTTTATTTTATATATTTGAGGTTGACCACATGACCTTAAGGATACATATAGACAGTAAACTGGTTACTACAGTGAAGCAAATTAACATATCTACCATCGTACATAGTTACATTTTTTTGTGTGACAGGAACAGCTAAAATCTACGTATTTAACAAAACTCCTAAAGACAATACATTTTTATTAACTATAGCCCTCATGATGTACATTAGATC

**cHS4**

GGGGAGCTCACGGGGACAGCCCCCCCCCAAAGCCCCCAGGGATGTAATTACGTCCCTCCCCCGCTAGGGGGCAGCAGCGACCGCCCGGGGCTCCGCTCCGGTCCGGCGCTCCCCCCGCATCCCGAGCCGGCAGCGTGCGGGGACAGCCCGGGCACGGGGAAGGTGGCACGGGATCGCTTTCCTCTGAACGCTTCTCGCTGCTCTTTGAGCCTGCAGACACCTGGGGGATACGGGGAAAAGGGGAGCTCACGGGGACAGCCCCCCCCCAAAGCCCCCAGGGATGTAATTACGTCCCTCCCCCGCTAGGGGGCAGCAGCGACCGCCCGGGGCTCCGCTCCGGTCCGGCGCTCCCCCCGCATCCCGAGCCGGCAGCGTGCGGGGACAGCCCGGGCACGGGGAAGGTGGCACGGGATCGCTTTCCTCTGAACGCTTCTCGCTGCTCTTTGAGCCTGCAGACACCTGGGGGATACGGGGAAAA
